# Minimal Invasive Pericardial Perfusion Model in Swine: A Translational Model for Cardiac Remodeling After Ischemia/Reperfusion Injury

**DOI:** 10.3389/fphys.2020.00346

**Published:** 2020-04-22

**Authors:** Stefanie Marek-Iannucci, Amandine Thomas, Roberta A. Gottlieb

**Affiliations:** Cedars-Sinai Medical Center, Smidt Heart Institute, Los Angeles, CA, United States

**Keywords:** myocardial remodeling, ischemia-reperfusion injury, therapeutic hypothermia, heart failure, pericardial irrigation

## Abstract

**Rationale:**

Adverse remodeling leads to heart failure after myocardial infarction (MI), with important impact on morbidity and mortality. New therapeutic approaches are needed to further improve and broaden heart failure therapy. We established a minimally invasive, reproducible pericardial irrigation model in swine, as a translational model to study the impact of temperature on adverse cardiac remodeling and its molecular mechanisms after MI.

**Objective:**

Chronic heart failure remains a leading cause of death in western industrialized countries, with a tremendous economic impact on the health care system. Previously, many studies have investigated mechanisms to reduce infarct size after ischemia/reperfusion injury, including therapeutic hypothermia. Nonetheless, the molecular mechanisms of adverse remodeling after MI remain poorly understood. By deciphering the latter, new therapeutic strategies can be developed to not only reduce rehospitalization of heart failure patients but also reduce or prevent adverse remodeling in the first place.

**Methods and Results:**

After 90 min of MI, a 12Fr dual lumen dialysis catheter was place into the pericardium via minimal invasive, sub-xiphoidal percutaneous puncture. We performed pericardial irrigation with cold or warm saline for 60 min in 25 female farm pigs after ischemia and reperfusion. After one week of survival the heart was harvested for further studies. After cold pericardial irrigation we observed a significant decrease of systemic body temperature measured with a rectal probe in the cold group, reflecting that the heart was chilled throughout its entire thickness. The temperature remained stable in the control group during the procedure. We did not see any difference in arrhythmia or hemodynamic stability between both groups.

**Conclusion:**

We established a minimally invasive, reproducible and translational model of pericardial irrigation in swine. This method enables the investigation of mechanisms involved in myocardial adverse remodeling after ischemia/reperfusion injury in the future.

## Introduction

We recently reported beneficial effects of hypothermia in a novel swine ischemia-reperfusion model (Marek-Iannucci et al., 2019). Because of repeatedly expressed interest in the model, we here present details of the methodology, the aim of this manuscript being to demonstrate the safety and reproducibility of this novel technique, in order to make it accessible to other research groups interested in studying molecular mechanisms of adverse myocardial remodeling after ischemia-reperfusion injury. Despite percutaneous coronary angioplasty, about one third of the patients with myocardial infarction (MI), will not achieve a thrombolysis in myocardial infarction (TIMI) flow grade 3, which can partially explain the increased incidence of chronic heart failure despite early intervention (Rochitte, 2008; Kaul, 2014; Bouleti et al., 2015; Dai et al., 2017). This phenomenon, known as no-reflow, is thought to represent microvascular damage caused by extended ischemia, leading to tissue loss and non-regeneration of the myocardium (Kloner et al., 1974; Rochitte, 2008; Kloner, 2011; Rezkalla et al., 2017; Shi et al., 2017). No-reflow increases adverse remodeling of the left ventricle after MI, leading to increased dilation, congestive heart failure and mortality with an overall worse outcome (Kloner et al., 1974; Rochitte, 2008; Dai et al., 2017). Furthermore, it amplifies myocardial stunning, reperfusion arrhythmia, microvascular obstruction and cardiomyocyte death and is held responsible for about half of the eventual infarction size (Hausenloy, 2012). Therapeutic hypothermia (TH) has been applied in various forms in animal studies, showing a reduction in no-reflow area and MI size (Erlinge et al., 2013; Erlinge et al., 2014; Dai et al., 2015; Dai et al., 2017; Rezkalla et al., 2017). Even components of inflammation have shown to be attenuated after TH application (Mohammad et al., 2017; Shi et al., 2017). Various hypotheses have been proposed to explain adverse remodeling after MI, yet the exact molecular mechanisms behind it remain poorly understood (Rochitte, 2008; Kloner, 2011; Schirone et al., 2017). One approach has been to study the influence of TH on the myocardium after MI. Although studies in small animals have yielded promising results, clinical trials were not as successful and human findings remain controversial (Erlinge et al., 2014; Herring et al., 2014; Erlinge et al., 2015). Possibly the models used in successful animal studies are not feasible in the clinical setting and the correct translational model has not been described yet. Systemic TH has been used in the clinical setting after resuscitation for decades, focusing primarily on neurologic outcome after cardiac arrest (Spiel et al., 2009; Haugk et al., 2011). More recent research focused on the direct effect of TH on the myocardium, but multicenter trials such as CHILL-MI and RAPID-MI-ICE failed to show significant reduction in infarction size (Erlinge et al., 2014, 2015; Mohammad et al., 2017). Nonetheless those trials showed a decreased incidence in heart failure. Methods used in the clinical setting include retrograde cold perfusion through the coronary sinus, endovascular cooling with special catheters, intraperitoneal cooling systems, cold saline infusions and topical cooling with cooling pads or blankets (Erlinge et al., 2014; Herring et al., 2014; Nichol et al., 2015; Kloner et al., 2017). This leads us in two possible directions regarding future studies: First, the right technique regarding TH application, implementable in the clinical setting has not been found yet (Herring et al., 2014). Second, it might be necessary to study the exact mechanism by which TH improves myocardial remodeling in animal models to design specific therapies targeting these pathways independently of the use of TH in the clinical field. Therefore, we designed a minimally invasive large animal model in swine, utilizing pericardial perfusion with cold saline after MI. The focus of our study is to investigate the influence of TH on myocardial remodeling on a molecular basis, with the goal of inhibiting the adverse effects of remodeling after MI and preventing chronic heart failure. Importantly, this model can be used for various other studies requiring direct application of substances or stimulation of the myocardium.

## Materials and Methods

### Animal Procurement

All procedures and protocols were approved by the Institutional Animal Care and Use Committee (IACUC) and were performed according to the National Institutes of Health (NIH) Guidelines for the Care and Use of Laboratory Animals. For the entire project, using a novel minimal invasive pericardial perfusion model in swine, 25 female farm pigs (35–40 kg) were ordered at S&S Farms (Ramona, CA, United States). The animals had a one-week acclimatization period prior to the procedure. They received food (Lab Diet-Porcine lab grower) twice a day and water *ad libitum*. Among all the 25 pigs, 6 animals died due to refractory arrhythmia within the first 45 min of MI, resulting in a survival rate of 76%. None of the animals died during the pericardial perfusion treatment or within the observation period until euthanasia, making the cause of death a common complication of ischemic injury of the myocardium, with no difference between the groups. Most of the data acquired with this model has recently been published by our group (Marek-Iannucci et al., 2019). The data in this manuscript consists in additional hemodynamic studies, supplementary to our prior publication, in order to demonstrate the reproducibility and safety of this method, the final goal being to make this novel method accessible to many other laboratories interested in studying molecular mechanisms of adverse myocardial remodeling after MI in a large animal model.

### Anesthesia

On the day of procedure, the pigs received sedating premedication with Ketamine (20 mg/kg IM), Acepromazine (0.25 mg/kg IM), Atropine (0.05 mg/kg IM), and Propofol (2.0 mg/kg IM). Two intravenous cannulas were placed and the animals were intubated. Continuous general anesthesia was maintained with Isoflurane 1–3%.

### Medication

Amiodarone 10 mg/kg was given continuously intravenous over 30 min in 250 ml of 5% Dextrose prior to intervention. 2% Lidocaine was given as continuous infusion (2 mg/kg/h) throughout the whole procedure to reduce the risk of arrhythmia. To prevent blood clotting in the catheters we administered two boli of intravenous heparin (100 IU/kg) once prior to intervention and once after pericardial catheter placement. We used intravenous Amiodarone (Bolus dose of 10 mg/kg given in a 1:10 dilution of 5% Dextrose) and Lidocaine (bolus dose 40–60 mg) to terminate arrhythmia if necessary. Phenylephrine 1 mg/ml was used in boli of 1 ml as needed in case of systolic blood pressure drops below 40 mmHg.

### Angiography and Myocardial Infarction

We performed one procedure per day, at the same time each day, to minimize possible differences due to circadian rhythm. At first, basal left ventricular function (LVF) was measured with echocardiography (GE, vivid 7, and M-mode long axis). Mean LVF was 88% and mean end-diastolic volume 58 ml. Vital signs were measured continuously throughout the procedure. Heart rate and rhythm were monitored with defibrillator patches and conventional electrocardiogram. Peripheral oxygen saturation was measured with pulse oximetry and blood pressure with a sphygmomanometer prior to intervention. We performed a left sided surgical cutdown of the neck, to access the left common carotid artery and the left common jugular vein. An 8-Fr and 7-Fr sheath were placed into the carotid artery and jugular vein respectively. The central venous access was used for continuous intravenous fluid infusion, blood collection and administration of medication. After successful placement of the arterial sheath, blood pressure was measured invasively. A coronary guide catheter was used for angiography. A 3.00 or 3.50 mm balloon (depending on the size of the coronaries, visualized in the angiogram) was then advanced into the left anterior descending artery and placed just distal of the first diagonal branch. The balloon was inflated until complete occlusion of the vessel and the pigs underwent 90 min of MI. All animals had to be shocked at least once with 200 Joules due to ventricular fibrillation but were otherwise stable throughout the MI. After 90 min the balloon was completely deflated and extracted under fluoroscopic control, followed by a 30 min period of reperfusion (see Table 1 for required material).

**TABLE 1:** List of the material required for the procedure.

List of material	
Description	Amount
12Fr dual lumen dialysis catheter set (Mahurkar-Elite, Medtronic^®^, Dublin, Ireland) includes all necessary tools for pericardial puncture	1/animal
Perisafe^®^ Weiss Epidural Needle (Becton Dickinson, Franklin Lakes, New Jersey)	Optional
7Fr and sheath for coronary angiography	1 (reusable)
8Fr and sheath for coronary angiography	1 (reusable)
3.0–3.5 mm angioplasty balloon	1 (reusable)
i.v. line perfusion set	2 (inflow and outflow)/animal
Sterile container to collect fluid	1/animal
Thermo-bag for infusion sets	1 (reusable)
Sterile saline for infusion	1–2 L/animal, depending of infusion rate

### Pericardial Puncture

During the first 30 min of reperfusion, we performed a sub-xiphoidal, and percutaneous pericardial puncture. We filled a 10 cc syringe with 5 cc sterile saline and attached it to the puncture needle provided in the catheter set. To reduce the risk of ventricular perforation it is possible to use a Perisafe^TM^ Weiss Epidural Needle (Becton Dickinson, Franklin Lakes, NJ, United States) instead. Under fluoroscopic imaging we performed a midline, percutaneous, sub-xiphoidal puncture, and 1–2 cm below the xiphoid. Under fluoroscopic imaging the needle was inserted in a 45 degrees angle and advanced for about 1 cm. After that the angle was reduced to 15–20 degrees and the needle was advanced directing toward the midline of the right clavicle of the animal (when performed from the left side of the animal). There was a small resistance when penetrating the diaphragm. Under continuous vision with fluoroscopy and while maintaining suction on the syringe, the pericardium was punctured. While doing so one can feel a loss of resistance and loose forward movement of the needle. This crucial moment can be visualized via fluoroscopy when observed carefully. It is important to aspirate after puncture to ensure correct positioning. Aspirating blood suggests ventricle puncture (bright red for the left and dark red for the right ventricle). In case of correct positioning of the needle, nothing or a minimal amount of clear fluid (pericardial fluid) can be aspirated. There can also appear a small air bubble in the syringe, suggesting correct positioning in the pericardium. After that the syringe is detached and a guidewire (supplied with the puncture set) is advanced through the needle. Keep the needle secured with one hand at all time to avoid displacement. While inserting the guide wire under fluoroscopic control you can observe the wire encircle the heart. Always insert the guide wire generously to avoid displacement (Supplementary Figure S1). After that, we removed the puncture needle while the wire remains in place. We then performed a 3–4 mm incision with a sterile disposable scalpel at the entry site of the guide wire. A 12Fr dilator (supplied with the puncture set) was inserted over the guide wire and advanced to the incision site. It is important never to insert a catheter or dilator into the tissue without having the end of the guide wire reaching out at the end of the latter and secured with one hand. The dilator is then inserted under simultaneous and cautious retraction of the wire into the pericardium. It is crucial that a sufficient length of guide wire always remains inside the pericardium to avoid displacement (about 10 cm on imaging screen). After that, the dilator is removed and the guidewire gently advanced more deeply. We then performed the same maneuver once again using a 12-Fr dual lumen dialysis catheter (Mahurkar-Elite, Medtronic^®^, Dublin, Ireland) (Supplementary Figure S2). Once the catheter is in place, one lumen of the latter can be opened and a 10 cc syringe filled with 5 cc sterile saline and 5 cc dye can be injected to confirm correct placement under fluoroscopy (Supplementary Figure S3). If positioned correctly, the catheter will lie in a crescent form at the border of the heart shadow. It is important that the catheter is inserted neither too low nor too deep, to avoid occlusion. The dye should spread widely over the whole surface of the ventricle (Supplementary Figure S3). Finally, the guide wire can be removed, and the catheter secured with one or two single sutures.

### Pericardial Perfusion

The catheter was connected to a closed tube system filled with sterile saline. One port was used as inlet and the other one as outlet. The inlet port was attached to a standard intravenous fluid administration set with sterile saline. The outflow was collected in a sterile collection bottle. We performed one hour of pericardial irrigation with an average infusion rate of 1250 ml/h (20.8 ml/min). The animals were overall stable throughout the entire pericardial irrigation period, meaning that the infusion rate can be increased if desired. In the first couple of minutes the outflow may appear pink, tinged with blood, although it should not be deep red or viscous, which would suggest active bleeding into the pericardium. Infusion rate can be controlled with a pump or a flow metering system. After one hour the pericardial irrigation was ended. The inlet port was closed first and the outlet port only after complete arrest of fluid outflow. Suction at the end of the procedure on the outlet port can minimize the amount of remaining fluid in the pericardium. During the whole perfusion period it is important to monitor heart rate and blood pressure continuously via the arterial sheath. A reduction in blood pressure can indicate cardiac tamponade. To avoid the latter, the flow rate should be increased gradually over the first 2 min. Finally, the catheter was cautiously removed after opening the sutures. There should not occur any bleeding after removal of the catheter.

### End of Procedure

The sub-xiphoidal incision site was cleaned and betadine ointment was used to avoid infection. Sutures were not necessary, due to the minimal size of incision. If necessary one steri-strip can be used to close the incision. Following the intervention, the venous and arterial sheath were removed and both vessels were ligated. The skin was closed using 4-0 absorbable Vicryl^∗^ sutures intracutaneously.

### Optimization of the Technique

We lost 3 pigs due to ventricular fibrillation refractory to defibrillation therapy which eventually ended in electro-mechanic dissociation (EMD), most likely due to tamponade. As a matter of fact, placing the 12F dialysis catheter inside the pericardium prior to MI was not well tolerated by the animals. After changing strategy and placing the dialysis catheter after 90 min of MI and during coronary reperfusion we had no more animal losses. Furthermore, we tried to add a temperature probe into the pericardium for continuous measurements during pericardial irrigation. Unfortunately, the measurements were extremely dependent of the position of the dialysis catheter and the probe itself, making the data not entirely reliable (Supplementary Figure S4). We therefore decided to measure left ventricular temperature with a specific thermocouple probe (ThermoWorks, American Fork, UT, United States), inserted under fluoroscopic control into the left anterolateral ventricular wall percutaneously (Supplementary Figure S5).

### Statistical Analysis

For all experiments with a single comparison, we applied a 2-tailed unpaired Student’s *t*-test. For multiple comparisons we performed a one-way ANOVA including a Tukey’s *post hoc* analysis. Results are represented by the mean and standard error for the mean (SEM). A *p*-value of less than 0.05 war regarded as significant. All data acquired with echocardiography and Swan-Ganz pressure measurements have been repeated 3 times per animal and the mean has been used for further analysis. Regarding the temperature and blood pressure measurements, these consist in continuous monitoring throughout the procedures and have been there for performed only once per animal for each given timepoint.

## Results

We used this pericardial irrigation model on 25 female farm pigs (35–40 kg) to cool the myocardium locally and study its influence on myocardial remodeling after MI. The data presented here is acquired from 6 out of the 25 animals and consists in additional information to previously published data (Marek-Iannucci et al., 2019) on this novel technique, in order to demonstrate its safety and make it accessible to other laboratories interested in studying molecular mechanisms of adverse myocardial remodeling after MI in a large animal model.

**FIGURE 1:**
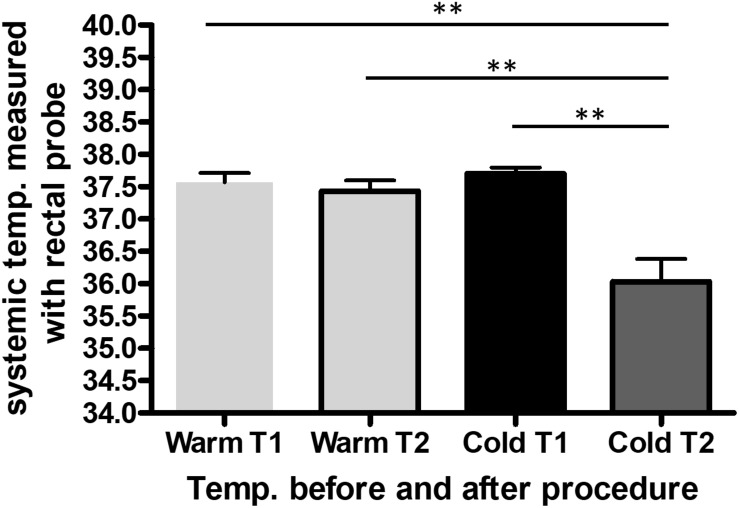
Average systemic body temperature (SBT) measured with a rectal probe throughout the procedure in the control (*n* = 3) and cold (*n* = 3) group. The data represents the systemic temperature before (T1) and after (T2) the entire procedure in the warm compared to the cold group. We performed one-way ANOVA, with ^∗∗^*p* < 0.01.

**FIGURE 2:**
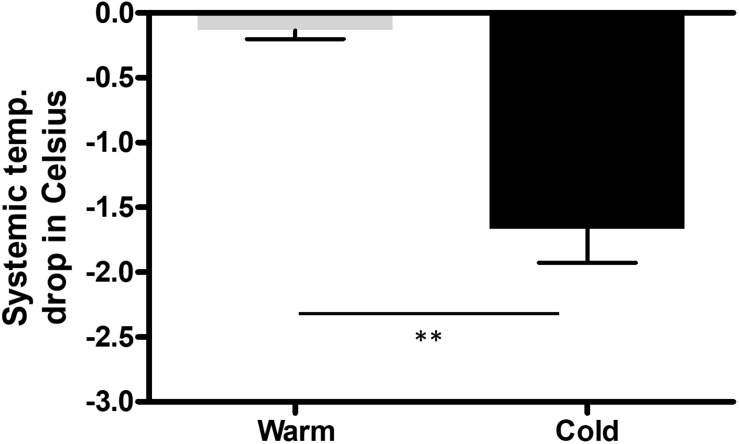
Change (ΔT) in systemic body temperature (SBT) measured with a rectal probe throughout the procedure in the control (*n* = 3) and cold (*n* = 3) group from beginning to end of procedure. We performed an unpaired, two tailed *t*-test, with ^∗∗^*p* = 0.0047.

The saline was either cooled in the fridge (8^°^C) or warmed in the heating cabinet (37^°^C) for 3 days. To keep the temperature of the saline bag constant we used a specific thermo-bag for infusion sets. In case of cold saline, we added a consistent amount of cool packs into the latter. In the control group, pigs underwent pericardial perfusion with 37^°^C sterile saline for 60 min and remained at a stable systemic temperature, measured by a rectal temperature probe, and throughout the procedure. After the complete procedure of 3 h, their mean body temperature was 37.4^°^C (SEM = 0.17), with a mean temperature variation (ΔT) of 0.1^°^C (SEM = 0.07) from beginning to end. The infusion temperature was measured at 37^°^C and the mean outflow of the pericardium was at 28.7^°^C. In the cold group, the pigs had a mean systemic temperature of 36.1^°^C (SEM = 0.35) with a mean drop of 1.7^°^C (SEM = 0.26) compared to the control group after one hour of cold pericardial irrigation. The mean inflow temperature was 11.5^°^C and the mean outflow 20.8^°^C. This can be optimized by adjusting the pericardial perfusion flow rate. The difference in mean systemic body temperature and ΔT were both statistically significant (*p* = 0.011 and *p* = 0.002 respectively) as shown in Figures 1, 2 respectively. We only needed to use phenylephrine in one animal with a blood pressure drop. During pericardial irrigation we did not encounter malignant arrhythmias. Overall, we did not encounter any differences in vital parameters or heart rhythm in the 2 groups (Table 2). The animals adapted well and we did not have any loss after the intervention. After one week the pigs were euthanized and the heart was harvested.

**TABLE 2:** Vital parameter measurements throughout the procedure.

		pre procedure NIBP	Incision NIBP	Sheath placement NIBP	Angiogram NIBP	Balloon inflation PB	Balloon deflation PB	Beginn irrigation PB	End irrigation PB	End procedure NIBP
17P49 Warm 1	HR	76	67	69	70	70	87	78	80	83
	RR syst	79	78	73	88	50	53	50	50	83
	RR diast	33	27	26	34	35	32	3i	32	30
	Sp02	98	100	99	98	98	100	100	100	100
17P54 Warm 2	HR	110	95	104	95	92	92	93	93	90
	RR syst	80	90	83	77	66	57	51	59	81
	RR diast	31	36	30	23	39	45	37	46	24
	Sp02	98	99	94	100	94	95	99	97	96
17P67 Warm 3	HR	82	75	73	54	65	68	66	68	73
	RR syst	62	80	72	69	54	60	53	60	79
	RR diast	20	37	31	31	36	42	38	22	22
	Sp02	97	96	95	96	96	100	100	100	100
17P62 Cold 1	HR	85	74	75	70	69	65	68	63	78
	RR syst	92	93	90	94	59	51	50	52	78
	RR diast	31	31	28	26	33	35	27	40	22
	Sp02	100	100	100	100	100	100	95	100	100
17P53 Cold 2	HR	92	77	76	74	73	68	68	66	72
	RR syst	86	97	101	54	59	50	51	45	87
	RR diast	17	24	24	33	38	32	33	28	16
	Sp02	98	99	98	98	33	98	98	96	98
17P66 Cold 3	HR	93	69	66	62	64	57	56	55	53
	RR syst	73	63	62	53	63	52	59	74	89
	RR diast	18	38	21	21	47	25	35	50	66
	Sp02	98	100	100	99	99	99	99	99	99

*Regarding blood pressure measurements, NIBP represent non-invasive blood pressure measurements on the extremities, whereas PB represents invasive pressure measurement in the ascending aorta throughout the procedure with the catheter. PB measurements are preferred, because more accurate, but not available throughout the entire time.*

**FIGURE 3:**
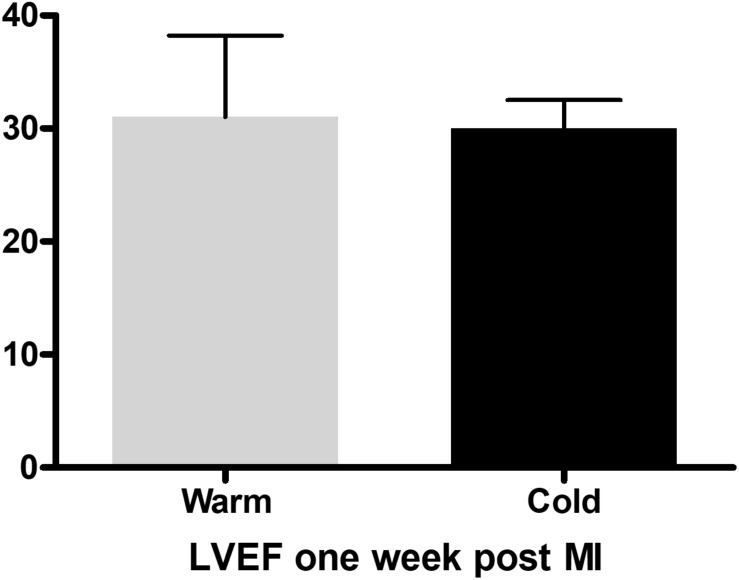
Ejection fraction measured with the Teichholz method in *M*-Mode one week after myocardial infarction in the control (*n* = 3) and cold (*n* = 3) group, with no difference between the groups, measured with an unpaired, two-tailed *t*-test.

Prior to euthanasia the pigs were anesthetized, and we performed echocardiography as well as ventriculography to assess LVF. We did not detect a significant difference in ejection fraction (EF) one week post MI (Figure 3). This is consistent with previously published data, where significant improvement of EF could be detected only beginning 4 weeks post MI (Marek-Iannucci et al., 2019). These beneficial effects of local hypothermia on EF were accompanied by a strong correlation between EF and effective cooling of the left ventricle as previously published (Marek-Iannucci et al., 2019). Furthermore, by using echocardiography our group published a significant increase in E′, a reduced E/E′ and left atrial pressure (LAP) in the cold group compared to the warm, suggesting improved diastolic function, when using this technique (Marek-Iannucci et al., 2019). With this method we were also able to demonstrate a significant reduction in Troponin I levels in the blood one week after MI in the cold compared to the warm group, as well as a reduction of neutral lipid droplets in the myocardium measured by electron microscopy, both reflecting a reduction in ischemic tissue injury as previously reported (Marek-Iannucci et al., 2019).

Blood analysis 3 h post MI showed a significant reduction of cardiac troponin I (*p* < 0.05); consistent with that, creatinine kinase and lactate dehydrogenase also trended lower in the cold (Figures 2E–G). Lastly, EM images of LV septal biopsies 3 h post MI showed a significant reduction of neutral lipid droplets (*p* < 0.05) correlating with tissue injury.

All animals had a minimal pericardial effusion with a maximal width of 1 mm without impairment of cardiac function. Furthermore, pressure measurements with a Swan-Ganz-catheter one week after survival were within the normal range in all animals (Table 3).

**TABLE 3:** Right heart catheter (Swan-Ganz-catheter) measurements one week after MI and prior to euthanizing the animals for tissue harvest.

	Syst *P*		Diast *P*	
	*mean*	*SEM*	*mean*	*SEM*
**Warm (*n* = 3)**				
RA	6.67	*0.33*	2.67	*1.33*
RV	22.33	*1.20*	5.33	*1.20*
Ao	63.00	*1.73*	37.67	*0.67*
**Cold (*n* = 3)**				
RA	6.33	*0.88*	3.00	*0.00*
RV	22.00	*1.53*	5.67	*0.67*
Ao	63.33	*1.76*	37.00	*5.51*

*The values represent pressure measurements in the right atrium (RA), right ventricle (RV), and the ascending aorta (Ao) in mmHg.*

## Discussion

To our knowledge this is the first local pericardial cooling model in a large animal. Dave et al. (1998) described a similar method in rabbits in 1998, with the disadvantage of requiring a specifically designed catheter for implementation. Furthermore, their group focused on myocardial infarct size reduction, without seeking to investigate the molecular mechanisms implicated in adverse remodeling after ischemia/reperfusion injury. Additionally, in their study the pericardium was perfused 30 min prior to infarction, making it a preconditioning model, which is not translatable to the clinical setting. Most prior studies have focused on treatment with hypothermia prior to, or few minutes after occlusion, and Hale et al. suggested that hypothermic treatment should be started prior to reperfusion to be effective (Hale and Kloner, 2011). TH has been studied in animals for decades and various models have been implemented. Kloner et al. developed a whole-body cooling method (Thermosuit^®^), with the capability of reducing the core body temperature to moderate hypothermia (Dai et al., 2015; Rezkalla et al., 2017; Shi et al., 2017). Other non-invasive models include cooling blankets, tested in rabbits, as well as convective immersion therapy (Herring et al., 2014). On the other hand, many invasive models have been established. Topical regional hypothermia was obtained with bags of iced saline, directly applied onto the outer surface of the heart in an open chest model in rabbits (Herring et al., 2014). Similarly, Dai et al. used a circuit to pump cold fluid directly into the thoracic cavity of rodents (Dai et al., 2017). Furthermore, studies showed the efficacy of retrograde blood cardioplegia and endovascular cooling, with a heat exchange balloon placed in the inferior vena cava in rodents (Kamlot et al., 1997; Herring et al., 2014). Other invasive approaches have been total liquid ventilation in Rabbits undergoing open chest surgery, as well as cold sterile saline infusion in swine (Herring et al., 2014), the latter being the only large animal model used for TH so far. From these studies we know that the time, temperature, and duration of TH application are crucial for the effects on the myocardium (Erlinge et al., 2013; Erlinge et al., 2014).

Our study is the first to prove that hypothermic treatment after coronary reperfusion is as effective as preconditioning or cold irrigation during ischemia, making this a clinically translatable model. This is demonstrated by the significant reduction of systemic temperature after the procedure, ensuring that the myocardium was affected in its entire thickness. The efficacy of this model can be underlined by a significant reduction of the temperature of the left ventricle, directly measured with a temperature probe inserted into the ventricle, as previously published (Marek-Iannucci et al., 2019). Furthermore, our previous study using this novel technique, demonstrated that Corrected TIMI frame count (CTFC), was significantly reduced after MI combined with pericardial irrigation in the cold compared to the warm group, with a strong correlation between ventricular cooling and CTFC, reflecting improved coronary reflow after MI (Marek-Iannucci et al., 2019). This is consistent with other studies demonstrating a beneficial effect of TH on the endothelium (Stamler et al., 1996). The fact that we cannot detect a difference in EF one week after MI is consistent with our previously published paper, as well as other studies, demonstrating that to detect substantial differences in ventricular function due to remodeling after MI, a longer follow-up (beginning at 4 weeks post MI) is required (Pokorney et al., 2012; Marek-Iannucci et al., 2019). In fact, by using this newly established large animal model, we showed significant beneficial effects on EF with local TH treatment four weeks after MI (Marek-Iannucci et al., 2019). Furthermore, by using this technique, were able to detect a significant reduction in local inflammation and fibrosis of the myocardium in the cold compared to the warm group, measured by immunohistochemistry with iNos, Arginase-1 and Picrosirius red staining respectively. Additionally, we observed a reduction in apoptotic cells in the myocardium of the cold group compared to the warm, as previously reported (Marek-Iannucci et al., 2019). Also, we previously demonstrated that this technique leads to a significant reduction of tissue stress markers in the myocardium, such as GRP78, HSP90, and HSP70, in the cold compared to the warm group, measured by western blotting (Marek-Iannucci et al., 2019). More importantly our previously published data demonstrates a significant increase in autophagic flux in the myocardium after cold pericardial irrigation, compared to the warm group, as well as a significant increase in spare respiratory capacity of the mitochondria, measured by respirometry. This suggests a more efficient cell renewal and more oxidative phosphorylation machinery per mitochondrion respectfully (Marek-Iannucci et al., 2019).

Furthermore, all pressure measurements, performed with a Swan-Ganz-catheter one week post MI, were within the normal range, demonstrating that the minimal amount of pericardial effusion remaining post procedure does not compromise contractile function, which was also confirmed by echocardiography (Kearns and Walley, 2018). Even if TH might not be directly implementable into the clinical setting with the existing animal models, it still remains an important tool to further study the underlying mechanisms of adverse remodeling after ischemia/reperfusion injury. The focus is to decipher possible new targets which can be therapeutically manipulated to reduce adverse remodeling and prevent progressive heart failure. The latter is known to be an independent prognostic factor for hospitalization and mortality, with an important socioeconomic burden for the health care system and remains a leading cause of death (Cohn et al., 2000; Wang et al., 2018). The introduction of Neprilysin-inhibitors is the first new substance group added to a well-established but stagnating therapeutic strategy for decades (McMurray et al., 2014). This underlines the necessity of further studies focusing on the molecular mechanisms behind adverse remodeling after ischemia/reperfusion injury, to broaden the therapeutic possibilities and eventually prevent progressive chronic heart failure.

To conclude, not only is our model very cost effective, with standard materials easily acquirable, it is also the first model in swine making it an important clinically translational model. It is well known that the physiology and coronary anatomy of swine very much resemble human. Furthermore, it is the first model that applied hypothermia to the heart after reperfusion with a long term survival. This model can be used for further studies on TH and its effects on myocardial adverse remodeling, as well as direct application of drugs or other agents influencing the latter.

## Data Availability Statement

All datasets generated for this study are included in the article/Supplementary Material.

## Ethics Statement

Animal studies were conducted under a protocol approved by the Cedars-Sinai Medical Center Institutional Animal Care and Use Committee (IACUC).

## Author Contributions

SM-I developed the model and performed the experiments. AT helped with the experiments and design of this manuscript. RG was the main PI and mentor of this project.

## Conflict of Interest

The authors declare that the research was conducted in the absence of any commercial or financial relationships that could be construed as a potential conflict of interest.

## Supplementary Material

FIGURE S1 Visualization of the guide wire used for pericardial puncture (provided with the dialysis catheter set).

FIGURE S2 Insertion on the dual lumen dialysis catheter (Mahurkar-Elite, Medtronic^®^, Dublin, Ireland), under continuous fluoroscopic control and consecutive injection of minimal amount of dye.

FIGURE S3 Injection of dye into the dual lumen dialysis catheter (Mahurkar-Elite, Medtronic^®^, Dublin, Ireland), under continuous fluoroscopic control to ensure correct placement.

FIGURE S4 Visualization of the dual lumen dialysis catheter (Mahurkar-Elite, Medtronic^®^, Dublin, Ireland), and the temperature probe inside the pericardium under continuous fluoroscopic control to ensure correct placement.

FIGURE S5 Visualization of the temperature probe, inserted percutaneously under fluoroscopic control into the left ventricle wall, allowing continuous temperature measurement.
