# Supplementary figures and images for: Minimal Invasive Pericardial Perfusion Model in Swine: A Translational Model for Cardiac Remodeling After Ischemia/Reperfusion Injury
Source: Front Physiol. 2020 Apr 22;11:346. doi: 10.3389/fphys.2020.00346 (PMC7188781; doi:10.3389/fphys.2020.00346)

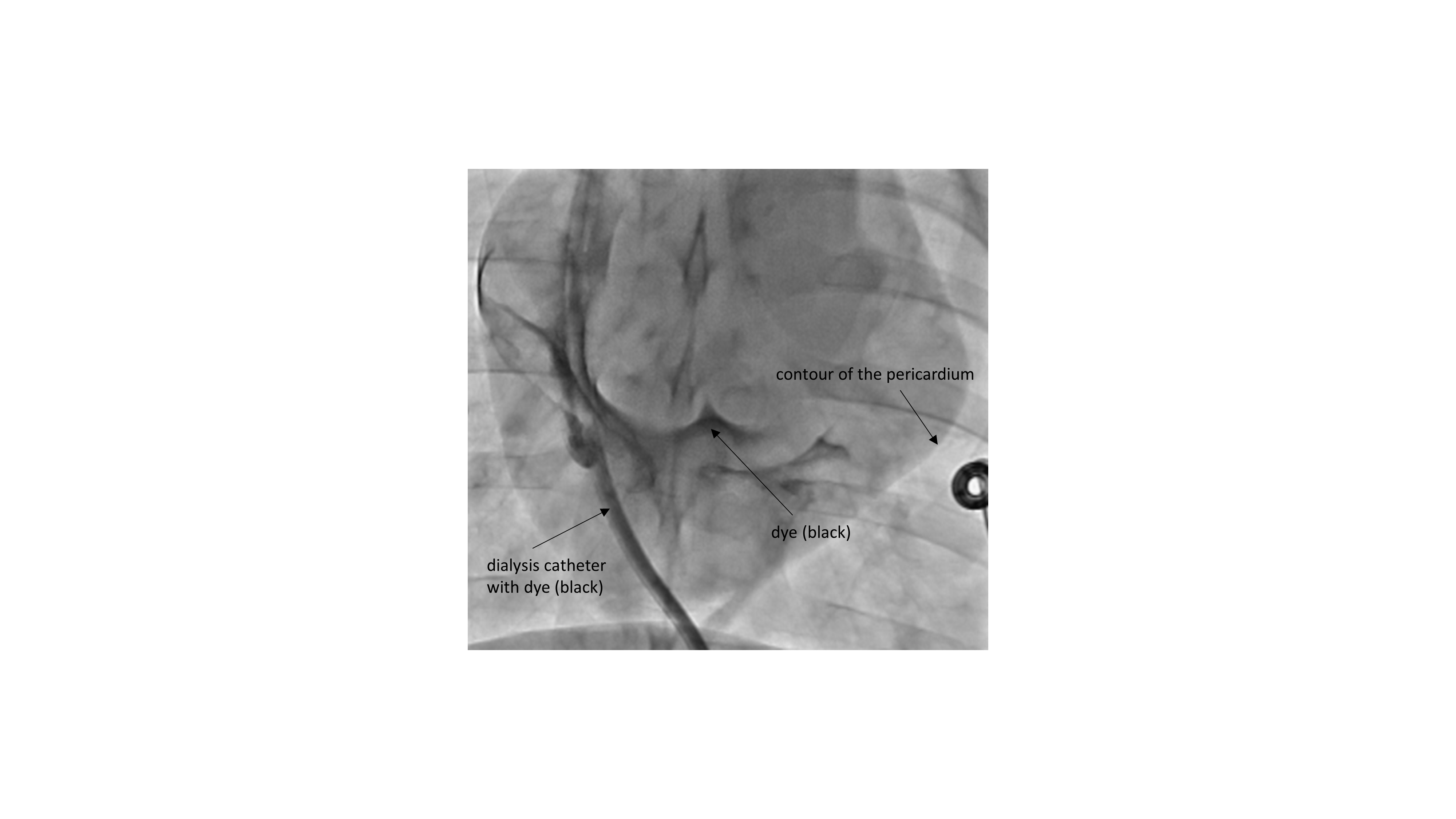

Supplement: FIGURE S1 — Visualization of the guide wire used for pericardial puncture (provided with the dialysis catheter set). [file Image_1.tif]

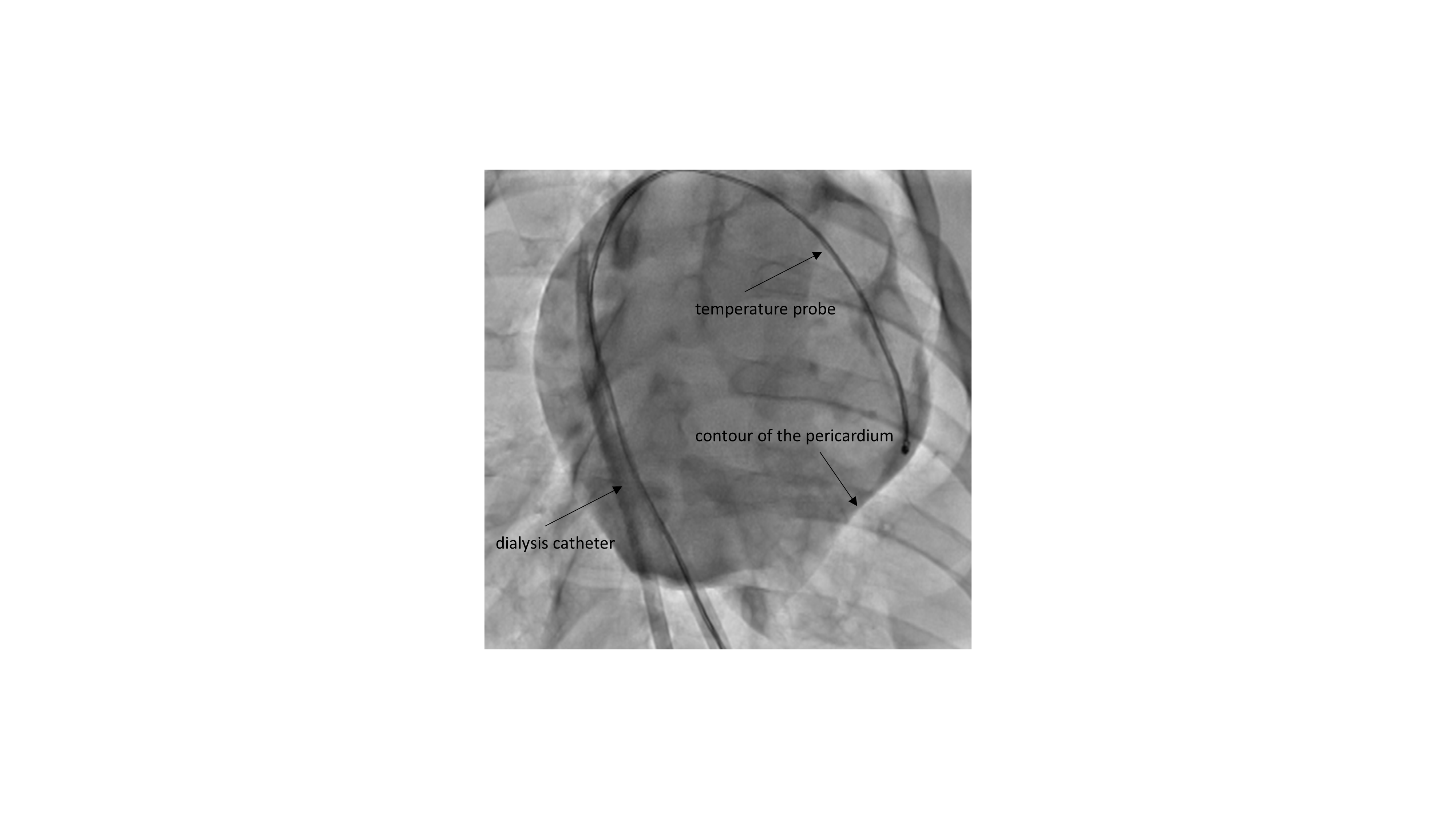

Supplement: FIGURE S2 — Insertion on the dual lumen dialysis catheter (Mahurkar-Elite, Medtronic®, Dublin, Ireland), under continuous fluoroscopic control and consecutive injection of minimal amount of dye. [file Image_2.tif]

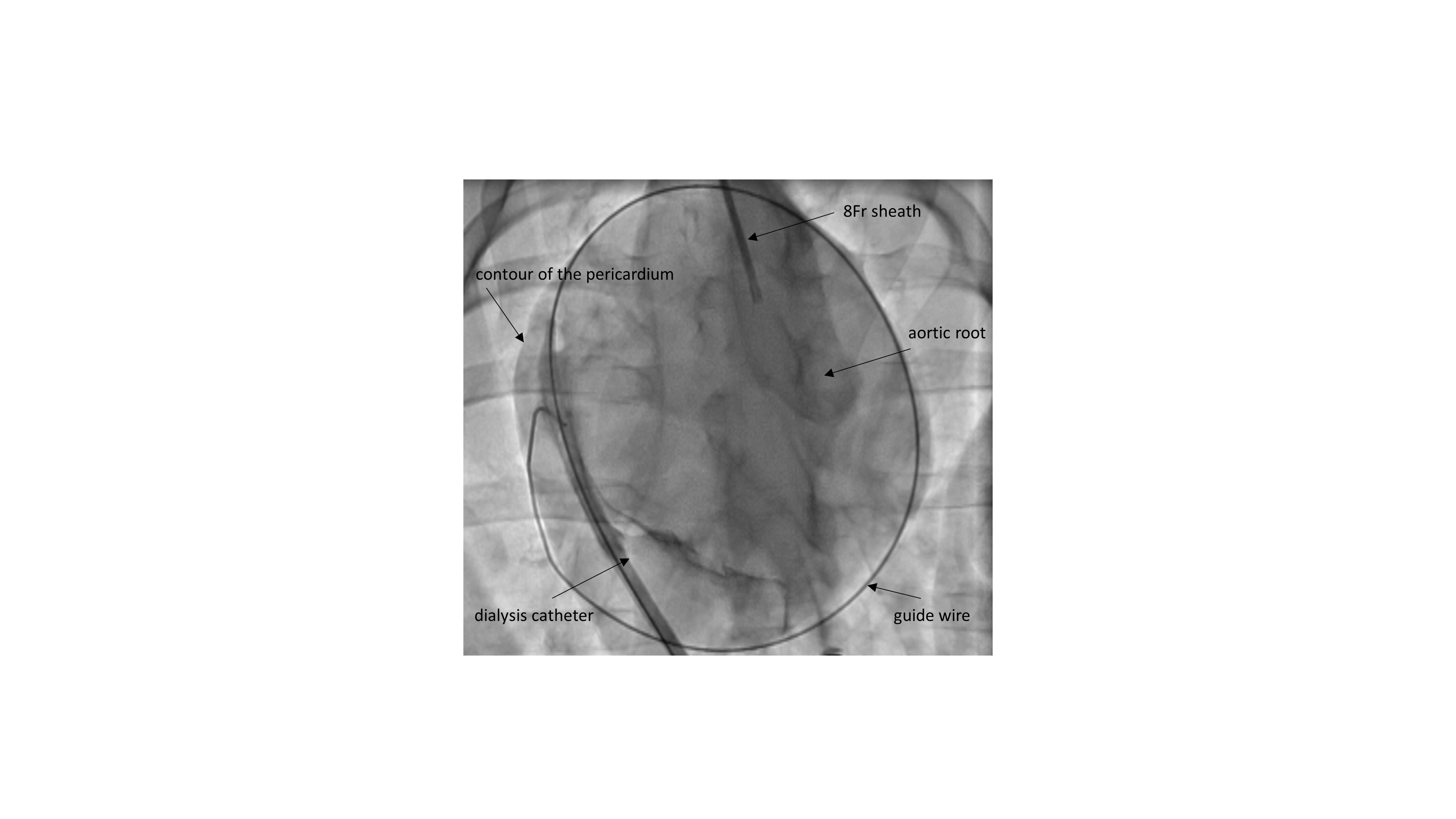

Supplement: FIGURE S3 — Injection of dye into the dual lumen dialysis catheter (Mahurkar-Elite, Medtronic®, Dublin, Ireland), under continuous fluoroscopic control to ensure correct placement. [file Image_3.tif]

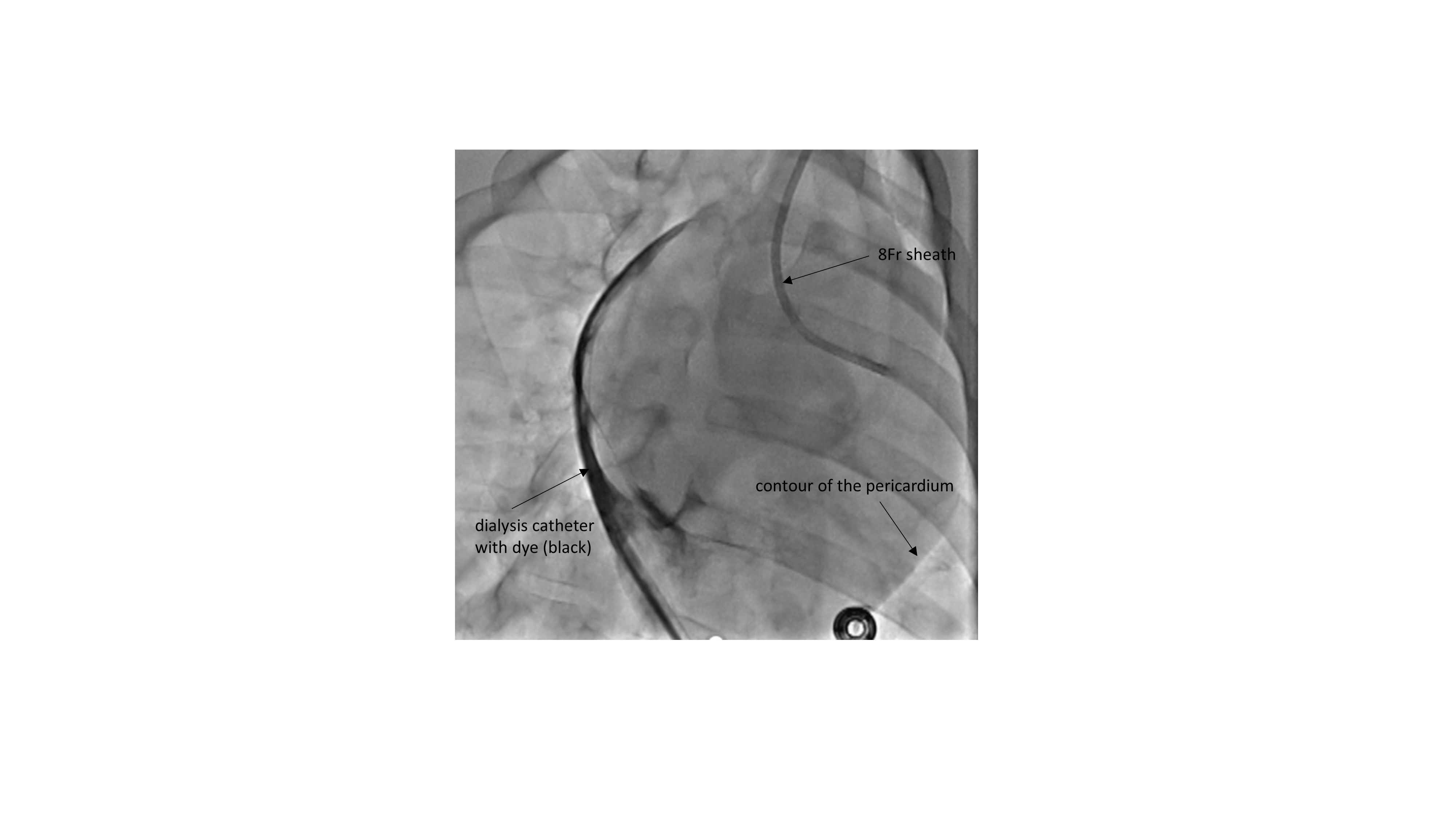

Supplement: FIGURE S4 — Visualization of the dual lumen dialysis catheter (Mahurkar-Elite, Medtronic®, Dublin, Ireland), and the temperature probe inside the pericardium under continuous fluoroscopic control to ensure correct placement. [file Image_4.tif]

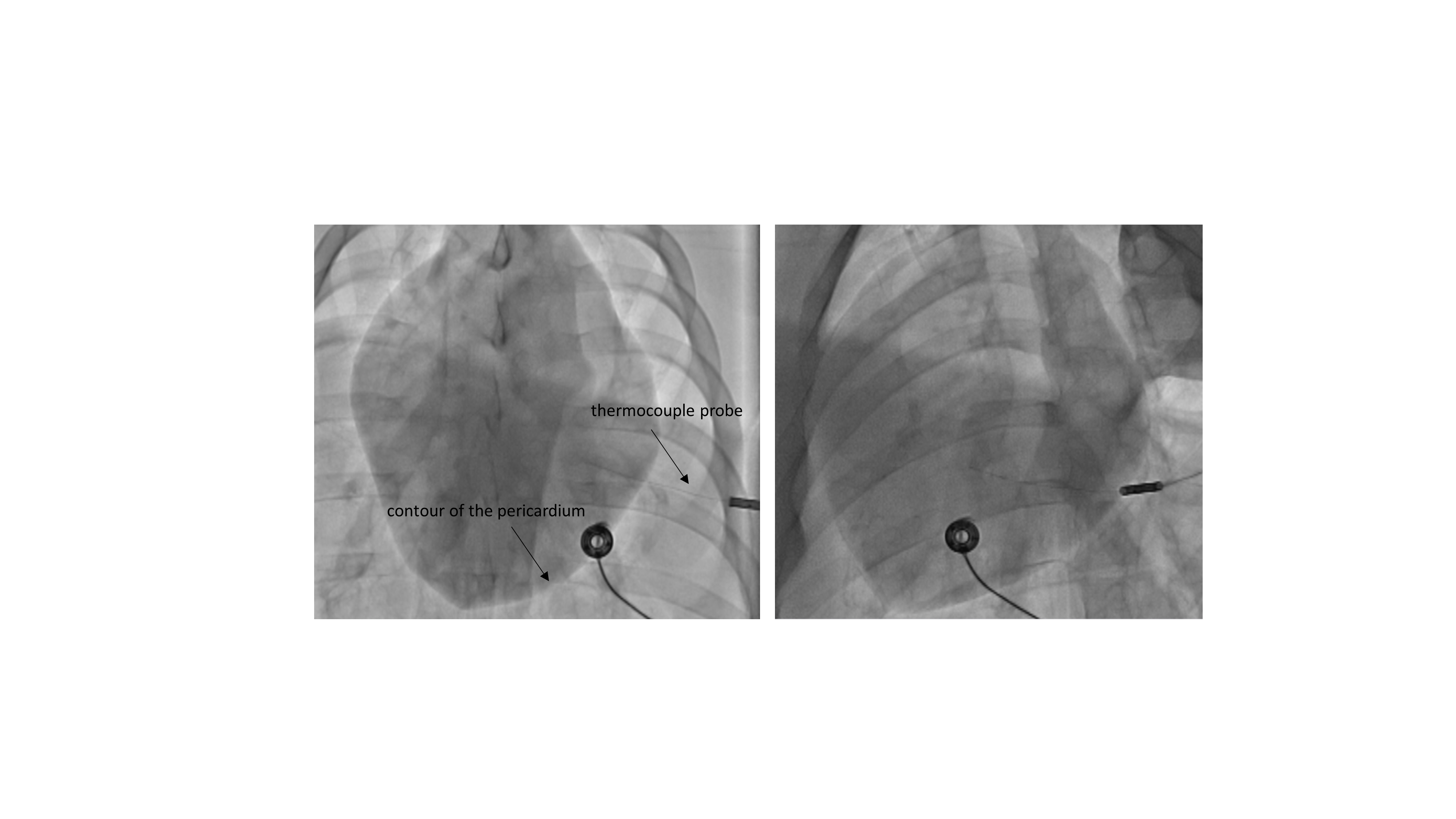

Supplement: FIGURE S5 — Visualization of the temperature probe, inserted percutaneously under fluoroscopic control into the left ventricle wall, allowing continuous temperature measurement. [file Image_5.tif]
